# Molecular and biochemical characterization of a potato collection with contrasting tuber carotenoid content

**DOI:** 10.1371/journal.pone.0184143

**Published:** 2017-09-12

**Authors:** Maria Sulli, Giuseppe Mandolino, Monica Sturaro, Chiara Onofri, Gianfranco Diretto, Bruno Parisi, Giovanni Giuliano

**Affiliations:** 1 ENEA, Casaccia Research Center, Via Anguillarese 301, Roma, Italy; 2 Scuola Superiore S. Anna, Piazza Martiri della Libertà 33, Pisa, Italy; 3 CREA-Centro Cerealicoltura e Colture Industriali, Sede di Bologna, Via di Corticella 133, Bologna, Italy; 4 CREA- Centro Cerealicoltura e Colture Industriali, Sede di Bergamo, Via Stezzano 24, Bergamo, Italy; University of Tsukuba, JAPAN

## Abstract

After wheat and rice, potato is the third most important staple food worldwide. A collection of ten tetraploid (*Solanum tuberosum*) and diploid (*S*. *phureja* and *S*. *chacoense*) genotypes with contrasting carotenoid content was subjected to molecular characterization with respect to candidate carotenoid loci and metabolic profiling using LC-HRMS. Irrespective of ploidy and taxonomy, tubers of these genotypes fell into three groups: yellow-fleshed, characterized by high levels of epoxy-xanthophylls and xanthophyll esters and by the presence of at least one copy of a dominant allele of the *β-Carotene Hydroxylase 2* (*CHY2*) gene; white-fleshed, characterized by low carotenoid levels and by the presence of recessive *chy2* alleles; and orange-fleshed, characterized by high levels of zeaxanthin but low levels of xanthophyll esters, and homozygosity for a *Zeaxanthin Epoxidase* (*ZEP*) recessive allele. Novel *CHY2* and *ZEP* alleles were identified in the collection. Multivariate analysis identified several groups of co-regulated non-polar compounds, and resulted in the grouping of the genotypes according to flesh color, suggesting that extensive cross-talk exists between the carotenoid pathway and other metabolite pathways in tubers. Postharvest traits like tuber dormancy and weight loss during storage showed little correlation with tuber carotenoid content, with the exception of zeaxanthin and its esters. Other tuber metabolites, such as glucose, monogalactosyldiacyglycerol (a glycolipid), or suberin precursors, showed instead significant correlations with both traits.

## Introduction

**Fig 1:**
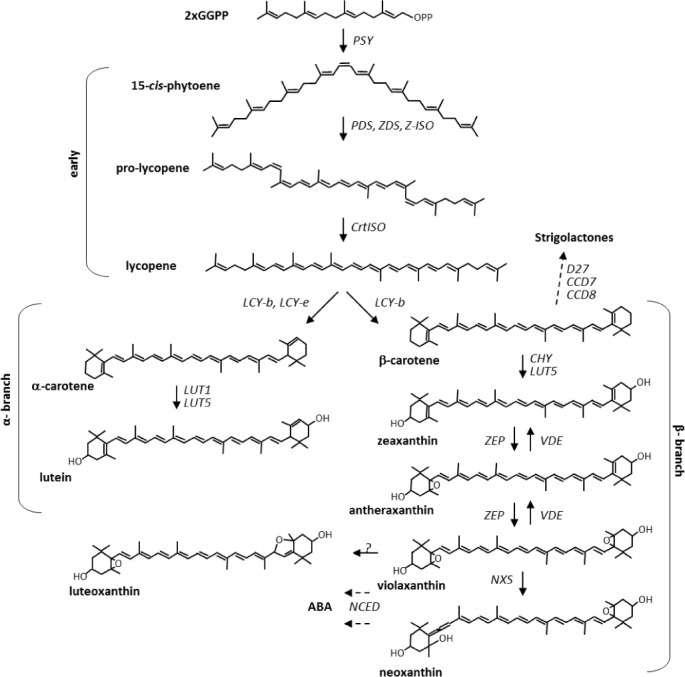
Schematic carotenoid biosynthetic pathway.

With a worldwide annual production higher than 300 million tons, potato (*Solanum tuberosum*) ranks third, after wheat and rice, as a staple crop for human nutrition (FAOSTAT 2013). Most early cultivated potatoes were diploid [1], while the greatest part of the commercial varieties are autotetraploid (2n = 4x = 48) [2], highly heterozygous and suffer from acute inbreeding depression. Tuber dormancy during storage represents an important trait for the fresh market, the processing industry, and seed tuber production [3] and is regulated by a range of physiological, environmental and hormonal factors, including abscisic acid (ABA). ABA is synthesized from the 9-cis-epoxycarotenoids neoxanthin and violaxanthin (Fig 1) *via* the intermediates xanthoxin and ABA-aldehyde. It has been suggested to be required for the establishment and maintenance of tuber dormancy [4].Potato tubers are a good source of starch, proteins, vitamin C and folate, but they generally show very low content of pro-vitamin A carotenoids [5] [6]. Carotenoids are C40 isoprenoid compounds that in plants are synthesized within the plastids [7]. They play essential roles in photosynthesis, while in non-photosynthetic tissues they exert a broad range of functions acting as pigments, and precursors of signaling molecules, including volatile ones [7]. Furthermore, they are essential dietary antioxidants and vitamin A precursors [8] [9]. Given the importance of vitamin A for human health, many metabolic engineering efforts have focused on the biofortification with β-carotene of important plant staples showing low provitamin A activity, such as rice, maize, wheat and potato [6][10–14].

The highest levels of total carotenoid in potato flesh are found in the groups *Phureja*, *Stenotonum* and *Goniocalyx* [15] [5] [16]. Two main loci have been shown to control potato tuber flesh color. The *Yellow* (*Y*) locus was characterized in crosses between Yema de Huevo (a diploid cultivar of the group Phureja) and diploid clone 91E22 (selected from crosses between groups Phureja and Stenotomum), which was shown to co-segregate with the *β-Carotene Hydroxylase 2* (*CHY2*) gene [16]. A dominant *Y* allele of this locus was associated with high flesh carotenoid content and showed increased expression of the *CHY2* transcript [17] [18]. The *Orange* (*Or*) locus was characterized in crosses between groups Phureja and Stenotomum and was associated with high amounts of flesh zeaxanthin [15]. The *Or* locus was found to co-segregate with the *Zeaxanthin Epoxidase* (*ZEP*) gene. All orange genotypes carry only a recessive *zep* allele (*zep1*) characterized by the insertion of a non-LTR retrotransposon in intron 1 and by reduced steady-state levels of the *ZEP* transcript.

In the present work, we performed a detailed characterization of a panel of ten potato clones, both tetraploids and diploids, and including a wild relative species (*S*. *chacoense*). Carotenoid content and type accumulated in tuber flesh were determined, as well the genotype and the transcript levels of the carotenoid-relevant loci (*CHY2* and *ZEP*), and the dormancy of the mature tubers. Extensive metabolomic profiling of the mature tubers was carried out for both polar and non-polar metabolites, and a series of bioinformatics approaches allowed to correlate carotenoid content with the general biochemical profile of the mature tubers, and their ABA and dormancy levels.

## Materials and methods

### Plant material and spectrophotometric carotenoid analysis

For the preliminary spectrophotometric determination of carotenoid content, we utilized tubers from 36 potato genotypes obtained from different sources. From the Julius-Kühn genebank, tubers of the following cultivars were obtained: Yema de Huevo, Filli, Gesa, Culpa, Keiblinger Karnten, Kranich, Liivi Collane, Monza, Omega and Urkartoffel; clones E 55/2, E 60/7, E 56/6, E 28/1, E 59/4, E 59/3, E 59/5, E 33/8, E 60/1 and cultivars Laura and Svenja were kindly provided by BNA (Germany); clone N 67/3 was provided by Norika (Germany); clone AG 22 and cultivars Andean Sunside and Papapura were from Agrico Research (The Netherlands); cv. Mayan Gold was obtained from the James Hutton Institute (Scotland); cv. Fontane was from Agrico B.A. (The Netherlands); tubers of the cultivars Kennebec and Majestic were provided by Cooperativa Produttori Sementi della Pusteria (Italy); clones ISCI 2/03-1, ISCI 1/12-3, ISCI 105/07-8 and cv. Melrose were from CREA breeding programs. Finally, *S*. *chacoense* accession GLKS 30919 was obtained from the International Potato Center (Peru). Tubers from genebanks were analyzed as such, while clones and commercial cultivars were analyzed after harvesting in the field at the CREA Anzola Emilia experimental farm. For spectrophotometric quantitation of carotenoids, lyophilized, homogeneously ground tuber samples (~0.2 g DW) were analyzed over two growing seasons as previously described [11].

### Metabolomics

Non-polar metabolites of selected potato clones (grown in 2012) were extracted from lyophilized, homogeneously ground tuber tissues and analyzed as previously described [19] [20] [21]-[22] with the following variations: the gradient was 0 to 1.2 min 95% A, 5% B; 3.5 min 80% A, 5% B; 12 min 30% A, 5% B, 65% C; 18 min 95%, 5% B. Chromatographic flux after equilibration was 0.8 ml/min and total run time 18 minutes. The atmospheric pressure chemical ionization-MS parameters were as follows: 40 units of nitrogen (sheath gas) and 20 units of auxiliary gas were used; the vaporizer temperature was 300°C, the capillary temperature was 250°C, the discharge current was 5.0 mA, and the capillary voltage and tube lens settings were 27 V and 95 V, respectively. Semi-polar metabolites were extracted from 20 mg lyophilized, homogeneously ground tuber tissue with 750 μl of 50:50 MeOH:H_2_O with 2 μM of reserpine as internal standard (Sigma Aldrich). Samples were shaken for 1h and, after centrifugation for 10minutes at 20,000 *g* at 4°C, 0.6ml of supernatant were transferred to HPLC filter tubes. 10μl of extract were injected to the LC-MS. LC analysis was performed using a C18 Synergi Hydro RP column (Phenomenex, Macclesfield, UK), 150x2.0mm, 4 μm particle size. Total run time was 40 min using an elution system consisting in A, water (0.2% formic acid, 10 pg/mL caffeine) and B, Acetonitrile:H_2_O 90:10 (0.2% formic acid, 10 pg/mL caffeine); the initial gradient was 95% A/5% B, followed by a ramp till 0%A/100%B in 30 min, before returning to the initial LC conditions in 4 minutes and an isocratic maintenance of 6 minutes. The MS analysis was performed as previously described [23] [24], but using the following parameters: capillary temperature 300°C, sheath and auxiliary gas set at, respectively, 50 and 5 units, spray voltage was 4.1 kV, capillary voltage was set at 46V and tube lens at 130 V. All the Chemicals and solvents used during the entire procedure were LC/MS grade (Chromasolv). Carotenoids were identified on the basis of their absorbance spectra (VIS), specific retention time (RT) and co-migration with authentic standards following the published method [19][25]. For quantification, all areas were normalized to the internal standard (DL-a-tocopherol acetate) and to their individual molar extinction coefficients [26]. A second normalization to a set of external standard was performed in order to calculate errors during injection in LC system. Carotenoid levels were expressed in terms of μg/gm of dry weight (DW). Other isoprenoids and semi-polar metabolites were identified based on their accurate masses (m/z), using both *in house* database and public sources (e.g. KEGG, MetaCyc, ChemSpider, LipidMAPS, PubChem), as well as co-migration with authentic standard, when available. The absolute intensities of each metabolite were relatively measured and expressed as fold on the internal standards, DL-a-tocopherol acetate or reserpine for non-polar and semi-polar metabolites, respectively. The measurements in the individual replicates are shown in S1–S4 Tables.

### Genotyping of the CHY2 and ZEP loci

The identification of the dominant allele 3 at the *CHY2* locus was performed by means of a specific CAPS assay developed by [18]. Briefly, the genomic DNA is amplified with primers CHY2ex4F (5’-CCATAGACCAAGAGAAGGACC-3’) and Beta-R822 (5’-GAAAGTAAGGCACGTTGGCAAT-3’) to obtain a 308 bp fragment extending from exon 4 to exon 5 of the *CHY2* gene. A subsequent AluI digestion gives rise to a diagnostic fragment of 163 bp in the presence of the dominant *Chy2* allele 3 whereas all the other (recessive) alleles at the same locus produce a specific fragment of 237 bp. The identification of the recessive allele 1 at the *ZEP* locus was based on the presence of a 4,102 bp retrotransposon insertion in the first intron of such allele. The surrounding genomic sequence was amplified with primers AWZEP25 (5’-CTGGCTGCATCACTGGTCAAAG-3’) and MSZEP26 (5’-GTGTAGTAGTCCTAGTCTTGCAC-3’), producing a 624 bp fragment in the presence of the retrotransposon, or with primers AWZEP25 and AWZEP20 (5’-TCATTCATAATTGTATCCTCCC-3’) which give rise to a 572 bp amplification product in its absence. The identification of the 49 bp deletion in the 4^th^ intron of *ZEP* alleles 1 and 10 was performed with a PCR assay as previously described [18], using primers AWZEP9 (5’-GTGGTTCTTGAGAATGGACAAC-3’) and AWZEP10 (5’-CACCAGCTGGTTCATTGTAAAA -3’).PCR amplification was performed with 1 U of Taq DNA polymerase (Promega), 1x reaction buffer, 200nM dNTP and 300 nM of each primer in a final volume of 50 μl. Standard amplification conditions were as follows: initial denaturation of 5 min at 95°C followed by 35 cycles of 30 sec denaturation at 94°C, 30 sec annealing at 55°C and 45 sec elongation at 72°C. Reactions were ended with an elongation step of 5 min at 72°C. The CAPS markers of the *CHY2* gene and the amplification products of the *ZEP* gene were separated on a 2% ethidium bromide-stained agarose gel.

### Cloning and sequencing of ZEP alleles 10 and 11

Two partially overlapping fragments at the 5’ end of the *ZEP* allele 10 found in Mayan Gold were amplified with primers ZEPFW4 (5’- AAATTACTACTCCACTAGTAGC-3’)/ ZEPREV1 (5’-TGGATCCTTTTCCGGAATAAGC-3’) and AWZEP25 (5’-CTGGCTGCATCACTGGTCAAAG-3’)/ AWZEP10 (5’-CACCAGCTGGTTCATTGTAAAA-3’), respectively. Both fragments span the retrotransposon insertion site of *ZEP* allele 1. Altogether they cover a 2304 bp region of the *ZEP* gene from position -358 relative to the transcription start site to the fifth exon. Amplification was performed using AccuPrime *Pfx*Taq DNA polymerase (Invitrogen) and cloned with the Zero Blunt PCR Cloning Kit (Invitrogen) according to the manufacturer’s instructions. Plasmid DNA from single colonies was isolated using the PureLink Quick Plasmid Miniprep Kit (Invitrogen). For each fragment, plasmid DNA from four independent clones was sequenced on both strands with M13forward and M13reverse universal primers. Sequencing was carried out at BMR Genomics (Padova, Italy) with an ABI 3730XL or ABI 3100 Sequencing device. A 2463 fragment of *S*. *chacoense zep* allele 11, extending from exon 1 to exon 7 of the *zep* genomic sequence was amplified with primers AWZEP25 and ZEPREV5 (5’-TGAGATGATATCCACAGGGC-3’). Amplification, cloning and sequencing were performed as for *zep* allele 10.

### Real time analysis of carotenoid biosynthesis genes

Total RNA was isolated from snap frozen ground tubers with Trizol Reagent (Life Technologies) according to manufacturer instruction for samples with high content of polysaccharides, starting from 200 mg of powdered tissue. One μg of RNA was then treated with DNase (Life Technologies) to remove any possible DNA contamination and retro-transcribed into cDNA with the High Capacity cDNA Reverse Transcription Kit (Applied Biosystems) following the kit indications.

Primers used to quantitatively detect mRNA expression of *Phytoene Synthases 1* and *2* (*PSY1* and *PSY2*), *Phytoene Desaturase* (*PDS)*, *β-Lycopene Cyclase* (*LCYb)*, *ε-Lycopene Cyclase* (*LCYe)*, *CHY2* and *ZEP* and of the housekeeping gene *Elongation factor 1α* (*EF1a*) were designed with the program Oligo Perfect Designer (Life Technologies) based on conserved intron-spanning regions of GenBank EST and cDNA sequences from *S*. *tuberosum* and PGSC genomic sequences from *S*. *phureja*. The primer sequences are shown in S5 Table. Equal amounts of cDNA (100 ng) from the previously described samples were used to measure the expression of carotenoid biosynthesis genes by Real time PCR in a RotorGene 6000 apparatus (Qiagen), using the Rotor-Gene SYBR Green PCR master mix (Qiagen) according to manufacturer instructions and *EF1a* as internal control gene. The primers used for the detection of the carotenoid biosynthesis gene and of *EF1a* levels were checked for comparable amplification efficiencies in serial dilutions of the cDNAs in order to analyze the data obtained through the relative quantification method of 2^-ΔΔCt^ [27].

### Tuber dormancy and weight loss measurements

For each genotype tested, at least six tubers grown in open field conditions and harvested at maturity were placed to sprout in the dark at room temperature, according to official protocols of NIAB (National Institute of Agricultural Botany, Cambridge, UK) and NPCF (Netherlands Potato Consultative Foundation, Den Hag, NL). Tubers were weighted twice: at the beginning of storage and after 100 days when dormancy was visually assessed. Weight loss during postharvest storage was expressed as a percentage of initial weight according to the following formula: % of weight loss: (W0 –W100)/W0 x 100 where W0 = initial weight and W100 = weight after 100 days of storage.

### Statistics and bioinformatics

For biochemical data analysis, ANOVA and Principal component analysis (PCA), the Past 3.11 software [28] was used in order to identify metabolites showing differential accumulation within the potato tuber collection. Heat-maps and Hierarchical Clustering Analysis (HCA) were applied using average linkage as agglomeration rule, to evaluate, respectively, the metabolic distribution patterns and the distance within genotypes, by using Genesis Software 1.7.6 [29]. Correlation analysis was performed using Pearson’s coefficient correlation [30]. Correlation network analysis was carried out using Cytoscape as previously described [12] [31] [32].

## Results

### Carotenoid and carotenoid ester contents of diploid and tetraploid potato tubers

**Fig 2:**
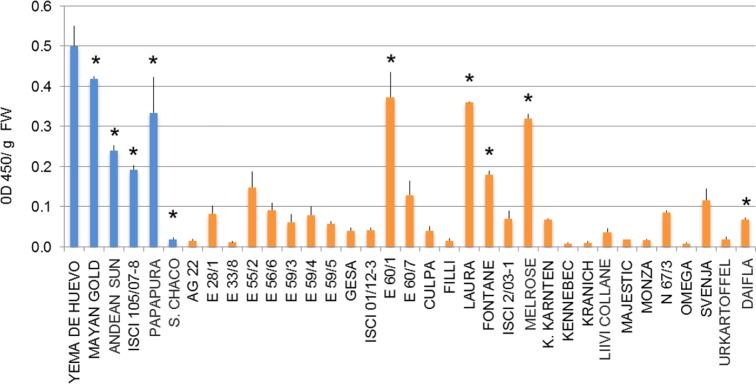
Total carotenoid content in mature tubers of a collection of potato genotypes. Tetraploid and diploid genotypes are depicted in orange and blue, respectively. Genotypes included in the core collection are marked with an asterisk. Data are the average ± stdev of 3 tubers.

**Fig 3:**
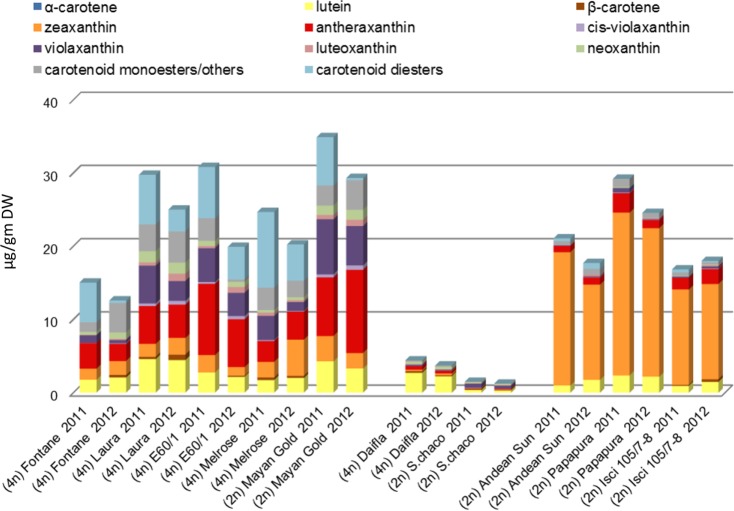
Carotenoid composition of mature tubers from 10 different genotypes grown over two different seasons, measured by LC-PDA-MS. Amounts of the different carotenoids are plotted as stacked bars. Data are the average of 3 biological replicates and are expressed as μg/gr DW. Detailed data are shown in S1–S6 Tables.

**Fig 4:**
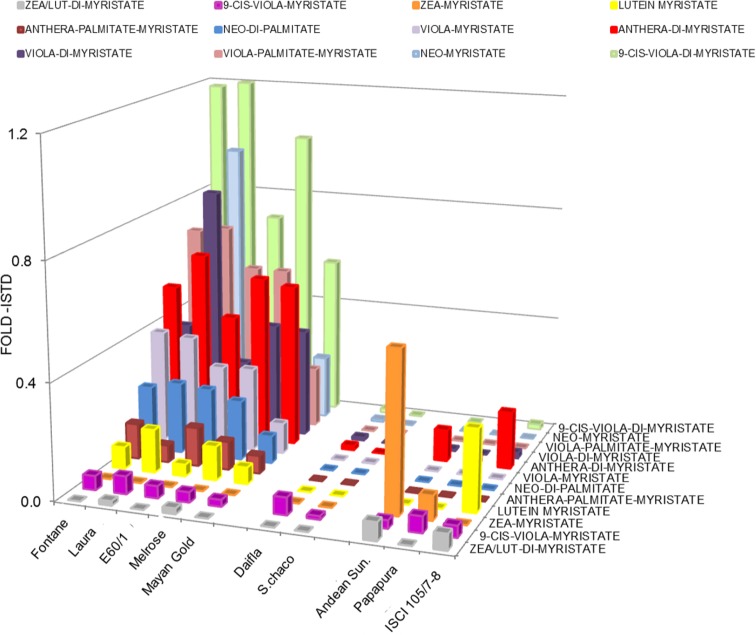
Levels of xanthophyll mono- and di-esters in potato tubers, measured by LC-PDA-MS. Levels are expressed as fold Internal standard (ISTD, α-tocopherol-acetate). Data are the avg from 3 biological replicates. Esters were identified as described in Materials and Methods (Δppm<2).

The tuber carotenoid content of a collection of 35 potato genotypes, both diploid and tetraploid, was measured by spectrophotometry (Fig 2). Tubers with high, intermediate and low carotenoid content were found in both diploid and tetraploid genotypes. A core collection of 10 genotypes with contrasting flesh color was selected for further analysis, including five tetraploid *S*. *tuberosum* (Fontane, Laura, E60/1, Melrose and Daifla), four diploid *S*. *phureja* (Mayan Gold, Andean Sunside, Papapura and ISCI 105/07-8) and one diploid *S*. *chacoense* clone. The high carotenoid genotypes comprised both yellow- and orange-fleshed clones. Carotenoid composition was determined by Liquid Chromatography—Photodiode Array online spectrometry -High Resolution Mass Spectrometry (LC-PDA-HRMS, see Materials and Methods). The results are shown in Fig 3 and described in S6 Table. Tubers could be grouped into three categories: a first group with yellow flesh and high levels of epoxy-xanthophylls (anthera-, viola- and neoxanthin) and xanthophyll esters, comprising tetraploid Fontane, Laura, E60/1, Melrose and diploid Mayan Gold; a second group (tetraploid Daifla and diploid *S*. *chacoense*) with white flesh and very low tuber carotenoid content; and a third group, with orange flesh and high levels of non-esterified zeaxanthin, which included diploid Papapura, Andean Sunside and ISCI 105/07-8. In contrast to what reported by [33], β-carotene levels were very low in all three groups. The relative levels of carotenoids among the potato genotypes analyzed were consistent over the two growing seasons (R^2^ = 0.9389) (S1 Fig). Xanthophyll esters were identified, based on their online PDA spectra and exact mass, including that of partially de-esterified fragments (Fig 4, S2 Fig and S7 Table), and quantified relative to the internal standard as described for other non-polar metabolites (See Materials and Methods). 23–41% of xanthophylls were present as esters in yellow fleshed genotypes, which contained mainly 9-cis-violaxanthin-dimyristate, antheraxanthin-myristate and violaxanthin-palmitate-myristate. In contrast, lower percentages (0.1–3.6%) of xanthophylls present as esters in orange-fleshed genotypes (Fig 4). 9-cis-viola-dimyristate was found at low levels in all genotypes, including white-fleshed ones, while zeaxanthin myristate was observed only in the orange-fleshed genotypes Andean Sunside and Papapura. Notably, all these esters contain only saturated fatty acids. Mass spectra were further searched in order to identify esters containing abundant mono- and poly-unsaturated fatty acids, such as eicosenoic, palmitoleic, linoleic and linolenic fatty acids, with negative results.

### Analysis of CHY2 and ZEP allelic composition

The core collection was genotyped at the *CHY2* locus using a CAPS (Cleaved Amplified Polymorphic Sequence) assay [18]. The results are shown in S3 Fig, and summarized in Table 1. The dominant *CHY2* allele 3, previously found to be a major determinant of carotenoid accumulation [16], was present only in yellow or orange-fleshed clones and its dosage was estimated: all genotypes with either yellow- or orange-fleshed tubers were heterozygous for allele 3, with the tetraploid clones bearing either 2 or 3 copies, but none being homozygous for such allele. Overall, no tight correlation between allele 3 copy number and total carotenoid amount was found. Homozygosity for the recessive *ZEP* allele 1 (*zep1*)—in the presence of at least one copy of *CHY2* allele 3- was reported to determine the orange-fleshed tuber phenotype, due to zeaxanthin accumulation. Two specific features distinguish allele 1 from the other *ZEP* alleles identified so far (all of which are dominant): a 4,102-bp retrotransposon insertion in the first intron, and a 49-bp deletion in the 4^th^ intron; this latter feature was the basis for the development of a PCR assay aimed at identifying its presence [18]. Based on this assay, we found homozygosity for *ZEP* allele 1 in the orange-fleshed varieties, as expected, but apparently also in the yellow-fleshed diploid cv Mayan Gold (see lane MG in S4 Fig, panel A), containing low levels of zeaxanthin (Fig 3). To solve this discrepancy, two complementary PCR assays, highlighting the retrotransposon insertion in the first intron, were used. The first yielded a band in the absence of the 4,102-bp element (upper lanes in S4 Fig, panel B) while the latter amplified a fragment spanning part of the retrotransposon sequence (lower lanes in S4 Fig, panel B). The results clearly showed that cv. Mayan Gold carried only one copy of the recessive allele 1 at the *ZEP* locus, together with a copy of another allele, with the same 49 bp deletion in the 4^th^ intron, but lacking the retrotransposon insertion, and acting as a dominant *ZEP* allele. Indeed, this allele determined the accumulation of antheraxanthin and lutein rather than zeaxanthin, and the consequent yellow color of Mayan Gold tuber flesh (Table 1). Furthermore, the *ZEP* allele of *S*. *chacoense*, represented by a variant fragment (S4 Fig, panel B, upper lane C) was identified as a putatively new allele. The partial sequencing of these new alleles was carried out, and the existence of these previously non-described variants was confirmed both in Mayan Gold (GenBank accessions JQ746499, *Zep* allele 10) and in *S*. *chacoense* (JQ746500, *Zep* allele 11).

**Table 1:** Allelic composition at the CHY2 and ZEP loci of the potato clones (shown in Fig 3). Detailed genotyping data are shown in S3–S5 Figs.

Genotype	Species	Ploidy	Genotype	
			*Chy2*:*chy2*	*Zep*:*zep*
Fontane	*S*. *tuberosum*	4n	02:02	04:00
Laura	*S*. *tuberosum*	4n	02:02	04:00
E 60/1	*S*. *tuberosum*	4n	03:01	04:00
Melrose	*S*. *tuberosum*	4n	03:01	04:00
Mayan Gold	*S*. *phureja*	2n	01:01	1*:1
Daifla	*S*. *tuberosum*	4n	00:04	04:00
GLKS30919	*S*. *chacoense*	2n	00:02	2**:0
Andean Sunside	*S*. *phureja*	2n	01:01	00:02
Papapura	*S*. *phureja*	2n	01:01	00:02
ISCI 105/07-8	*S*. *phureja*	2n	01:01	00:02

*Novel *ZEP* allele from Mayan Gold (GenBank: JQ746499).

**Novel *ZEP* allele from *S*. *chacoense* (GenBank JQ746500).

The diagnostic SNPs present in the 584 bp *ZEP* sequence between primers AWZEP9 and AWZEP10 -either known or new- are listed in S5 Fig; the relative dosage of dominant *ZEP* and recessive *zep* alleles in the potato germplasm examined is shown in the last column of Table 1.

### Expression of carotenoid biosynthesis genes

**Fig 5:**
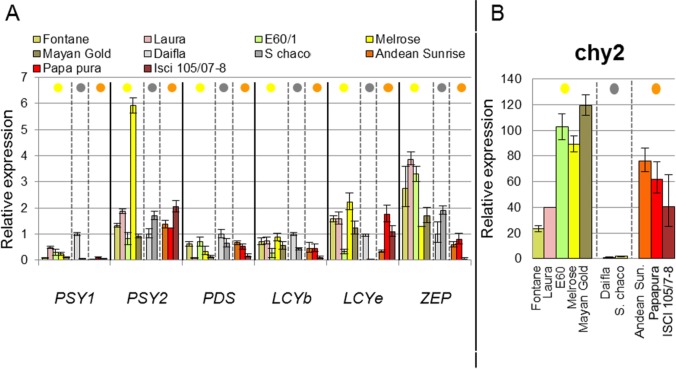
Transcript levels of carotenoid genes in mature potato tubers, measured by real time PCR. Data were normalized on the housekeeping *EF1α* gene, and on the white-fleshed cv. Daifla, used as calibrator (fold change = 1). **A**—*PSY1*, *PSY2*, *PDS*, *LCYb*, *LCYe*, *ZEP*. **B**—*CHY2*. For details, see Materials and Methods. Colored spots on top represent tuber flesh color (yellow, white, orange).

Transcript levels of several genes involved in carotenogenesis (*Phytoene Synthase 1 (PSY1)*, *PSY2*, *Phytoene Desaturase* (*PDS)*, *β-Lycopene Cyclase* (*LCYb)*, *ε-Lycopene Cyclase* (*LCYe)*, *CHY2* and *ZEP*, which catalyze the reactions represented in Fig 1, were measured in mature tubers of the core collection by Real Time quantitative RT-PCR. All gene expression data were first normalized on the housekeeping *EF1α* gene and then and on the expression level in cv. Daifla (Fig 5, S8 Table). The results showed some clear trends: *PSY2* steady-state transcript levels were generally higher than those of *PSY1*, in accordance with previous data [34]. *PSY2* expression was comparable in yellow, white and orange genotypes, with the exception of the yellow-fleshed cultivar Melrose, which exhibited significantly higher *PSY2* transcript levels than all other cultivars, with no impact, however, on total carotenoid content. With the exception of Melrose, the relatively similar *PSY2* transcript levels in a germplasm otherwise very different in terms of tuber carotenoid content, confirmed that transcription of this gene is not rate-limiting for carotenoid accumulation in tubers. Similarly, transcription levels of *PDS*, *LCYb* and *LCYe* did not show any significant relationship with carotenoid accumulation. On the contrary, a strong variation in *CHY2* expression was observed, reaching levels from 23- to 102- fold higher than white-fleshed Daifla in yellow-fleshed cultivars Fontane and Mayan Gold, respectively. In general, only a partial relationship was found between *CHY2* transcript levels and total carotenoid content of diploid and tetraploid yellow- and orange-fleshed clones. The variation of the *ZEP* transcript level was much lower than that of *CHY2*, ranging from 1.7-fold less (Andean Sunside), up to a maximum of 3.9-fold more (Laura). Diploid orange-fleshed clones, mostly accumulating zeaxanthin, exhibited the lowest levels of *ZEP* transcript, confirming its inverse relationship with the amount of zeaxanthin in potato tubers.

### Metabolic profiling

**Fig 6:**
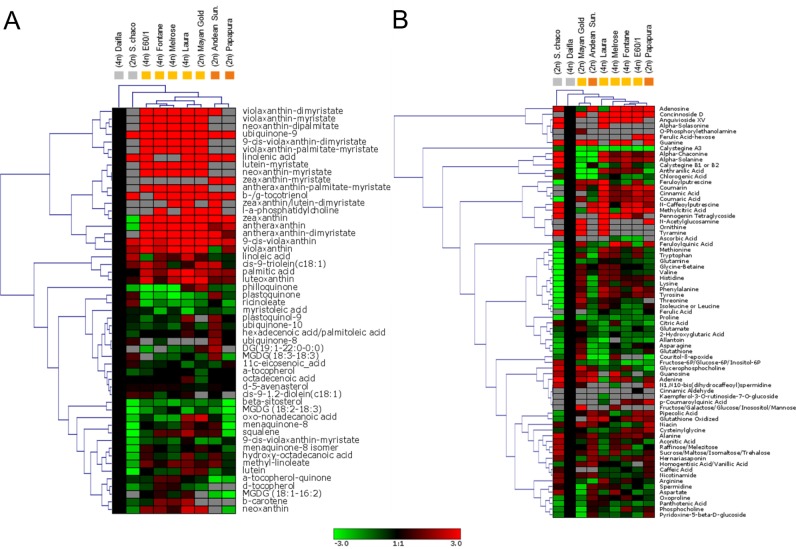
**Heatmaps of non-polar (A) and semi-polar metabolites (B) from potato tubers.** Colors represent metabolite levels in each clone normalized for the white-fleshed Daifla clone. Colored squares under the genotype name represent flesh-phenotype (orange, yellow, white). Red and green squares indicate up- and down-regulated metabolites, respectively. Fold-change values were log2-transformed. Both columns (genotypes) and rows (metabolites) were subjected to hierarchical clustering analysis (HCA).

**Fig 7:**
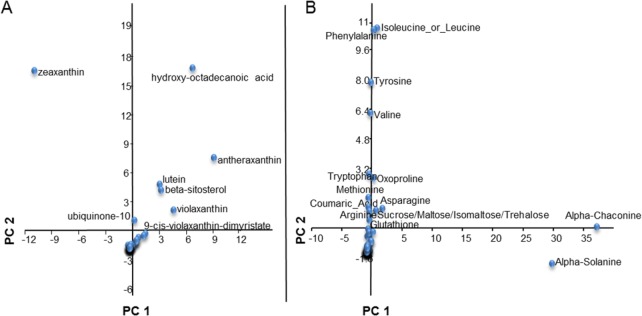
**Principal component analysis (PCA) showing the major discriminating non-polar (A) and semi-polar metabolites (B).** The first two principal components (PC1 and PC2) explained more than 98% of the total variance in both fractions.

The relative levels of 53 non-polar (free fatty acids, sterols, esterified carotenoids, tocopherols and quinones) and of 73 semi-polar metabolites (amino acids, amines, organic/phenolic acids and esters, nucleotides and nucleosides, peptides, polar lipids, sugars, polyols and phosphates, vitamins, amides, alkaloids and saponins, and phenylpropanoids) were measured using LC-HRMS. Metabolites were identified on the basis of accurate masses and retention times, using both in-house and public (e.g. Metlin, Kegg, MetaCyc, LipidMAPS) databases. A subset was validated using authentic standards. The metabolite levels, expressed as fold internal standard (ISTD, alpha-tocopherol acetate and reserpine for, respectively, the non-polar and semi-polar fractions), and normalized on the basis of the dry weight (DW), are shown in S9 and S10 Tables (non-polar and semi-polar, respectively). Overall, variation in metabolite levels was higher for the semi-polar than for the non-polar metabolome, with particular regard to alkaloids, organic acids, phenylpropanoids and vitamins. Contrary to what observed for carotenoids, genotypes showing similar flesh color did not exhibit similar patterns of accumulation for any of the other metabolite classes. Hierarchical clustering analysis (HCA) was applied to both the non-polar and semi-polar metabolites (Fig 6). In the non-polar HCA, yellow-, white- and orange fleshed clones group in three different clusters, indicating that composition of the non-polar metabolome is strongly associated with carotenoid composition. In both the non-polar and semi-polar HCA, tetraploid clones cluster together, indicating a possible selection for metabolic composition occurred either during polyploidization or during the domestication process. In order to identify the main metabolites responsible for the variance within the potato collection, Principal Component Analysis (PCA) was applied to both fractions (S6 Fig). As expected, the genotypes separated nicely by flesh color in the non-polar PCA than in the semi-polar one. This separation is mostly driven by carotenoids, in particular zeaxanthin, which showed the highest contribution to the orange- versus yellow-flesh separation, while antheraxanthin and lutein contribute to the separation of yellow- and white-clones along the first component (Fig 7). Interestingly, a fatty acid derivative, hydroxy-octadecanoic acid (m/z 299.2577, [M+H]+), also showed a high contribution to this separation. This is a consequence of the fact that this compound shows low levels in white-fleshed and high levels in yellow-fleshed genotypes. On the other hand, in the semi-polar PCA, *S*. *chacoense* was well separated from all the other clones, and this separation was driven by high levels of glycoalkaloids (alpha-solanine and alpha-chaconine) and low levels of free amino acids (isoleucine, leucine, phenylalanine and tyrosine) in this species (S6 Fig, Fig 7). Finally, high levels of sucrose or its isomers (maltose, trehalose and raffinose) were also detected in *S*. *chacoense* (S10 Table), confirming the peculiar semi-polar metabolome of this wild species.

### Postharvest phenotypes

**Fig 8:**
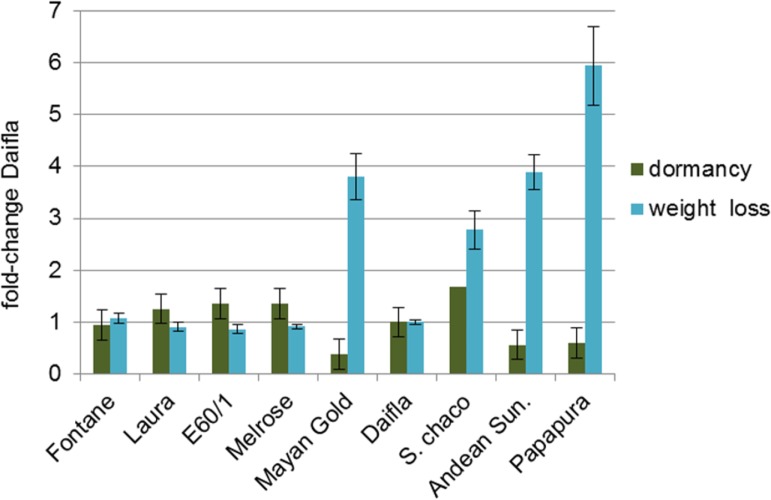
Post-harvest traits. Dormancy (green bars) and percent weight loss (light blue bars), measured on tubers dark-stored at room temperature for 100 DAH (Days After Harvest). Values are normalized for Daifla.

One of the major factors influencing potato quality is tuber dormancy, which negatively affects sprouting and weight loss during storage. Both dormancy and percent of weight loss after 100 days of storage were measured (S11 and S12 Tables; Fig 8). The highest level of dormancy was observed in white-fleshed diploid *S*. *chacoense*, with a score of 9.0, while other diploid genotypes (Andean Sunside, Papapura, ISCI 105/7-8 and Mayan Gold) showed low dormancy (score range 2.0–3.2), in accordance with their *Phureja* background [35]. Yellow-fleshed clones showed medium to medium-high dormancy scores. Regarding weight loss, *S*. *chacoense* showed intermediate levels, while other diploid genotypes showed high levels and tetraploid clones showed low levels.

### Correlation analyses between metabolic and phenotypic traits

The Pearson’s correlation coefficient ρ [30] was used to evaluate correlations within traits, metabolites and transcripts showing statistically significant differences between the white fleshed Daifla and other samples. To this end, all values were made non-dimensional by normalizing for Daifla, and then Pearson’s ρ values were computed for each trait pair, which values are showed as correlation matrix in S13 Table. Monogalactosyldiacyglycerol (MGDG 18:1–16:2), a glycolipid, was negatively correlated with weight loss (ρ = -0.94) and positively with dormancy (ρ = 0.80). To the opposite, glucose (a hexose), p-coumaroylquinic acid (a hydroxycinnamic acid derivative) and tyramine (a tyrosine derivative), showed strong negative correlations with dormancy and positive with weight loss, respectively. Although carotenoids did not show strong correlations with dormancy (all ρ values being <|0.75|), weight loss correlates positively with zeaxanthin (ρ = 0.77).

**Fig 9:**
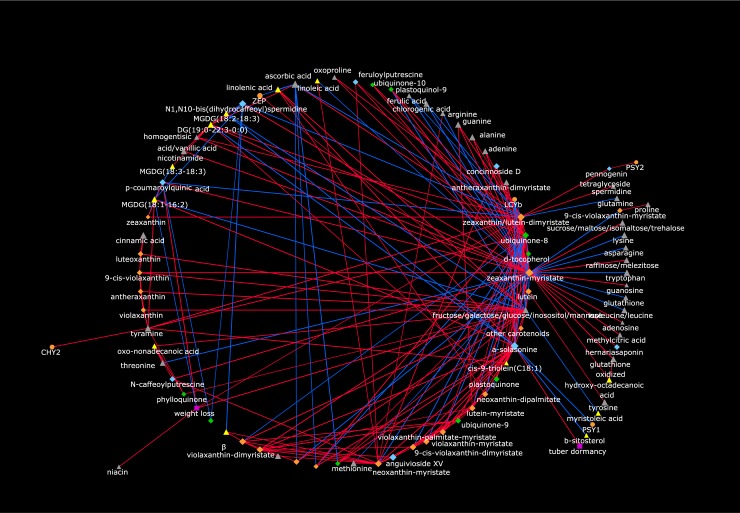
Correlation network of carotenoid transcripts, metabolites and post-harvest traits. Pearson correlation (r) coefficients were generated by using fold change-Daifla values. Node shape is according the kind of data (transcript: round; primary metabolite: triangle; secondary metabolite: diamond; post-harvest phenotype: square). Node colors identify compounds belonging to different pathways (semi-polar primary metabolite: gray; non polar primary metabolite: yellow; carotenoid: orange; semi-polar secondary metabolite: light blue; isoprenoid: green) or a post-harvest phenotype (violet). Node size is proportional to node strength (ns). Red and blue edges refer to, respectively, positive and negative correlations. Edge thickness is according the corresponding |ρ|. Only ρ > |0.90| are shown. Regions populated by nodes displaying a large number of significant positive and negative correlations are magnified.

In order to better visualize the “global” relationships between metabolites, carotenoid genes and post-harvest traits, we drew a correlation network based on the previously described correlation matrix (S13 Table). The network (Fig 9) displays only correlations whose ρ ≥ |0.90|. The strengths of each node in the network, defined as ns = Avg|ρ| [12], are shown in S13 Table and are proportional to the size of the corresponding symbol. Interestingly, most correlations were of positive sign, suggesting the overall dataset, specifically primary and secondary metabolites, is tightly co-regulated within the potato collection under study. Furthermore, it was possible to identify, on the rightmost part of the network, a region populated by a series of nodes with either a high ns or a high number of strong (ρ ≥ |0.90|) correlations towards other nodes: these include polar primary compounds (adenine, guanine, ascorbic acid, fructose or isomers), one quinone (ubiquinone-8), one glycoalkaloid (a-solasonine) and, notably, several carotenoid esters (such as neoxanthin-dipalmytate, zeaxanthin-myristate, zeaxanthin/lutein-dimyristate), which constituted the most represented metabolite class in this highly correlated region. Additional carotenoid esters, like violaxanthin-dimyristate, antheraxanthin-palmitate-myristate, 9-cis-violaxanthin-dimyristate and violaxanthin-palmitate-myristate, also displayed high ns values, and are shown in the lower part of the network.

## Discussion

The genetic control of carotenoid content in potatoes has been primarily studied in segregating diploid populations [15] [16] [17] [18]. These studies showed that: i) the *Y* locus, conferring yellow or orange tuber flesh, co-segregates with the *CHY2* gene; ii) a dominant *CHY2* allele is associated with increased *CHY2* expression and with high levels of xanthophylls in tubers; iii) the *Or* locus, conferring orange flesh, corresponds to the *ZEP* gene; a recessive *zep* allele is associated with decreased *ZEP* steady- state levels and increased zeaxanthin content; and iv) *CHY2* is epistatic to *zep*, consistent with the fact that the former gene acts upstream of the latter in the carotenoid pathway (Fig 1).

The data presented in this paper confirm these previous observations. The mechanisms whereby the *CHY2* and *ZEP* genes control tuber carotenoid accumulation are relatively straightforward. *CHY2* is probably rate-limiting for xanthophyll production, and its overexpression allows an increased accumulation of antheraxanthin, lutein and violaxanthin, the main xanthophylls accumulated by yellow-fleshed tubers, consistent with the fact that *CHY2* acts in both the β-xanthophyll (anther- and viola-) and lutein pathways. Carotenoid content was higher in genotypes containing at least one copy of the dominant *CHY2* allele 3 [16] [18] [36]. Accordingly, all the yellow- and orange-fleshed cultivars analyzed in the present work were heterozygous for *CHY* allele 3 (Table 1). In the wild species *S*. *chacoense*, homozygosity for a new *CHY2* allele was observed; since this *S*. *chacoense* allele was associated with a white-fleshed tuber phenotype, we assume it is functionally equivalent to the already described recessive *chy2* alleles (e.g. alleles 1, 2 and 5, Table 1).

Homozygosity for the recessive *zep* allele 1 resulted in lower *ZEP* gene expression, zeaxanthin accumulation and orange-fleshed tubers. Zeaxanthin accumulation was observed only in genotypes in which homozygosity of *zep* allele 1 was combined with at least one copy of the dominant *CHY2* allele 3. We also identified a new *ZEP* allele 10 in Mayan Gold, carrying the 49-bp indel in the 4^th^ intron, but not the retrotransposon insertion in the first intron. Except for the retrotransposon insertion, this allele was identical, over a 2,352 bp region sequenced, to recessive *zep* allele 1, but acted as a dominant allele, resulting in high *ZEP* expression and low zeaxanthin content. This suggests that *ZEP* allele 10 is the progenitor from which *zep* allele 1 originated by insertion of a retrotransposon in the first intron, and that the retrotransposon insertion is the cause of the recessive loss-of-function in ZEP leading to the accumulation of zeaxanthin. Given the impact of the *ZEP* gene not only on zeaxanthin levels but on the whole tuber carotenogenesis [34] [37], it is of utmost importance to unambiguously distinguish, in breeding programs, the various *ZEP* alleles. For instance, [36] described a diploid population (03TR2) of *S*. *tuberosum* Group Phureja segregating for tuber flesh color where apparently *zep* allele 1 had no effect on zeaxanthin accumulation, concluding that in 03TR2 other genetic factors override the effect of *zep* allele 1. In view of our findings, the most likely explanation is that 03TR2 contains the *Zep* allele 10 we identified in Mayan Gold, or another dominant allele carrying the same 49 bp deletion, that is not able to cause zeaxanthin accumulation in the homozygous or heterozygous states. Since the *zep1* PCR assay carried out by [18] and [36] was based on the detection of the 49-bp deletion in the 4^th^ intron, it is possible that *zep* allele 1 frequencies reported in these papers need to be reassessed with more informative PCR assays (S4 Fig, Panel B). Besides Mayan Gold allele 10, another *ZEP* allelic variant was found in the wild species *S*. *chacoense* (*ZEP* allele 11, see Table 1 and S5 Fig) containing a 46-bp insertion in the first intron. Although allele 11 is found in a homozygous condition in *S*. *chacoense*, the lack of the dominant allele 3 at the *CHY2* locus prevented the clarification of its effect on zeaxanthin accumulation. Whatever the case, the new *CHY* and *ZEP* alleles described in this work suggest that the allele diversity at these loci in potato germplasm is very high.

Based on LC-HRMS analysis of tuber carotenoids, the clones examined were grouped into three classes, according to their flesh color. The yellow-fleshed group was characterized by the prevalence of antheraxanthin as the main free carotenoid, followed by violaxanthin and lutein. Zeaxanthin on average accounted for 10% of the total carotenoid fraction in this group, as reported also by other authors [33], while β-carotene fraction was negligible, never exceeding 3% of total carotenoids. The orange-fleshed group was characterized by a high carotenoid content (16–29 μg/g DW), composed mainly of zeaxanthin, which ranged from 74% (ISCI 105/7-8) to 86% (Andean Sunrise in 2011); furthermore, orange-fleshed varieties showed very little carotenoid esterification (see below). The white-fleshed group consists of only a 4n variety (Daifla) and a 2n *S*. *chacoense* with small white tubers. These two genotypes exhibited a markedly different HPLC carotenoid profile, as *S*. *chacoense* accumulated 3-times less carotenoids than cv Daifla and had a higher proportion of violaxanthin vs. total carotenoids than any other genotype. On the contrary, the main carotenoid present in cv. Daifla was lutein (Fig 3).

In yellow-fleshed tubers, a high proportion of the xanthophylls was partially esterified with the saturated fatty acids myristate (C14:0) and palmitate (C16:0), a fact that may positively contribute to their stability [38] and negatively to their bioavailability [39]. Occurrence of xanthophyll esters in potato tubers has been reported by other authors [36] [40] [41]. The types of xanthophyll esters we found match closely those found by [40], with the addition of several myristate monoesters, such as zeaxanthin myristate (Andean Sunside) lutein myristate (ISCI 105/7-8), neoxanthin myristate (Laura) and lutein myristate (several yellow-fleshed genotypes). Interestingly, in spite of the fact that we searched extensively for esters containing mono- or polyunsaturated fatty acids, we were not able to identify any, even if the parent fatty acid was abundant in the free form (eicosenoic, palmitoleic, linoleic and linolenic acid). This may indicate that xanthophyll acyltransferases mediating xanthophyll esterification have an absolute requirement for saturated fatty acids as their substrates. A putative acyltransferase mediating xanthophyll esterification with myristic and palmitic acids in tomato flowers has been recently cloned [42] and study of its mechanism of action will clarify this point.

Despite the fact that isoprenoid metabolites use the same biosynthetic precursor of carotenoids, i.e. GGPP (Fig 1), our metabolic profiling data do not support a simple competition model between carotenoids and other isoprenoids for GGPP, in the sense that the levels of non-carotenoid isoprenoids do not show a simple inverse correlation with those of carotenoids. This is particularly evident for quinones, in which some (plastoquinol-9, ubiquinone-9) are positively correlated with carotenoids, while others (plastoquinone, ubiquinone-8) show negative correlations with carotenoids, with the exception of zeaxanthin and its esters (S13 Table). Individual nutritionally relevant metabolites show different trends in the analyzed genotypes. For instance, delta-tocopherol (vitamin E) is low or undetectable in the orange genotypes, while phylloquinone and menaquinone-8 (vitamin K) are reduced in *S*. *chacoense* and Papapura. With regard to semi-polar metabolites, besides the high levels of anti-nutritional compounds such as glycoalkaloids (alpha-chaconine and alpha-solanine) in *S*. *chacoense*, ascorbic acid (vitamin C) and several antioxidant flavonoids are highly accumulated in Daifla. Although the genetic determinants controlling the biosynthesis of these compounds in potato tuber are still poorly understood, these data constitute an indication that, with the exception of beta-carotene (provitamin A), a large variability is available in domesticated germplasm for breeding for biofortification with multiple micronutrients.

In our core collection, we observed a strong negative correlation between dormancy and weight loss during storage. This suggests that these two traits are at least partially controlled by the same set of factors, acting in opposite ways. The negative correlation between monogalactosyldiacyglycerol and weight loss is coherent with the view that membrane lipids modulate postharvest water loss [43]. Similarly, both hydroxycinnamic acids and tyramine are essential building blocks of suberin, a hydrophobic polymer that constitutes the main barrier against water loss in many plant organs, including the potato tuber [44] [45], and that their increased levels in some genotypes may reflect decreased suberin deposition. It has been proposed [46] that the initial steps of sprouting probably rely on pre-existing soluble sugars (including glucose) rather than to mobilization of complex carbohydrates such as starch. This could explain the strong negative correlation we observe between free glucose and tuber dormancy.

No major correlations were found between the levels of any of the carotenoids and tuber dormancy. The only significant correlations were those of zeaxanthin, and of its myristate diester, with weight loss during storage. Although zeaxanthin is an indirect precursor of ABA, through violaxanthin and neoxanthin (Fig 1), the data do not support an involvement of ABA in the control of tuber weight loss: first, because violaxanthin and neoxanthin do not show any strong direct or inverse correlation with this trait (S13 Table), and second because ABA content itself does not show strong correlations with weight loss or tuber dormancy in our core collection. Thus, our data do not support the idea that ABA levels play a major role in the control of tuber dormancy, in agreement with other authors [47] [46].

## Conclusions

In this work, a complete genotyping of the two most relevant structural loci for carotenoid accumulation, *CHY2* and *ZEP*, and a thorough metabolomics analysis of mature tubers were carried out in a core collection of ten varieties, representing the known natural variation in carotenoid content in cultivated potato genotypes. New alleles at the *ZEP* locus were identified, in particular *Zep10* from the yellow-fleshed diploid cv. Mayan Gold, was shown to be the likely dominant progenitor of the recessive *zep* allele 1, without the retrotransposon insertion responsible for the orange-fleshed phenotype. Carotenoid accumulation in the germplasm tested showed a good consistency across two growing season analyzed, with regard to both total amount and type of carotenoids accumulated. However, carotenoid esters, present in considerable amounts in yellow-fleshed varieties, were accumulated in a season-dependent manner. We observed a large variability in the non-polar and semi-polar metabolomes, suggesting the absence of direct relationships between carotenoid and the primary and secondary metabolic pathways under study. No clear correlation was found between carotenoid content and postharvest attributes (dormancy and % weight loss), providing clues about the existence and additional regulative mechanisms not yet elucidated in potato tuber physiology. Correlation network visualization highlighted the relevant role covered by the carotenoid ester group in the metabolomics fluctuations of the potato tuber collection under study, although the precise basis of this role will need a more detailed investigation.

## Supporting information

S1 Fig Total carotenoid amounts over two different harvests. Carotenoids were measured by LC/PDA/HRMS analysis, and expressed as μg/gr DW. Symbols are colored according to tuber flesh color (orange, yellow, white).
(EPS)

S2 Fig Identification of xanthophyll mono- and di-esters in potato tubers. Esters were identified on the basis of their PDA spectra, accurate mass and chemical formula, calculating xanthophylls mono-/di- esters accurate masses, using a mass error < 2ppm. Peaks were identified as (a) neoxanthin- (b) violaxanthin- (c) 9-cis-violaxathin-myristate, (d) violaxanthin-palmitate-myristate.
(EPS)

S3 Fig CAPS marker analysis for the presence and dosage of the dominant CHY2 allele 3. A 308bp PCR product from the amplification of the Chy2 gene with primers CHY2ex4F and Beta-R822 was digested with AluI. Allele 3 gives rise to a specific 163 fragment (lower arrow) while all the other (recessive) alleles produce a 237 bp fragment (upper arrow).
Legend: I = ISCI 105/07-8; A = Andean Sunside; P = Papapura; MG = Mayan Gold; E = E60/1; L = Laura; F = Fontane; Mel = Melrose; D = Daifla; C = *S*. *chacoense*.
(EPS)

S4 Fig Assays for Zep alleles. Panel A: Determination of the presence of the recessive allele zep1 based on a PCR assay exploiting the 49-bp deletion in zep1 (see Materials and Methods). Amplification with the primer pair AWZEP9/AWZEP10 reveals alternatively the presence of zep1 alleles (535 bp fragment) or of any of the dominant Zep alleles (584 bp fragment). Legend as in S1 Fig., Y = 2n cv. Yema de Huevo. Panel B: Determination of the presence of the recessive allele zep1 based on a PCR assay exploiting the absence (primers AWZEP25/AWZEP20, upper lanes) or presence (primers AWZEP25/MSZEP26, lower lanes) of the retrotransposon characterizing the known zep1 alleles. Note the presence of fragments in both cases in the cv. Mayan Gold, and the variant fragment in *S*. *chacoense* (lane C). Legend as in the S1 Fig.
(EPS)

S5 Fig SNPs found in alleles 10 and 11, in cv. Mayan Gold and in *S*.*chacoense*. The SNPs positions are compared with all other known ZEP alleles (1–9, Wolters et al. 2010). The region sequenced was obtained as described in Material and methods and in S1 Fig.
(EPS)

S6 Fig **2D Principal Component Analysis grouping potato genotypes according to (A) non-polar and (B) semi-polar metabolite composition.** Samples are colored according the different flesh colors. The first two principal components (PC1 and PC2) explained more than 85% of the total variance.
(EPS)

S1 Table Carotenoid composition of different potato genotypes over two different growth seasons, determined by LC-PDA-HRMS. Data represent normalized levels, expressed in term of μg/gr of Dry Weight (DW), of three biological replicates (1–3) analyzed over two growing seasons. Averages (AVG) and standard deviations (ST DEV) are further indicated.
(XLSX)

S2 Table Levels of non-polar metabolites in potato genotypes measured by LC-APCI-HRMS. Data represent normalized fold-ISTD values of three replicates (1–3). Averages (AVG) and standard deviations (ST DEV) are further indicated.
(XLSX)

S3 Table Levels of semi-polar metabolites in potato genotypes, measured by LC-APCI-HRMS. Data represent normalized fold-ISTD values of three replicates (1–3). Averages (AVG) and standard deviations (ST DEV) are further indicated.
(XLSX)

S4 Table Levels of xanthophyll mono- and di-esters in potato genotypes measured by LC-APCI-HRMS. Data represent normalized fold-ISTD values of three replicates (1–3). Averages (AVG) and standard deviations (ST DEV) are further indicated.
(XLSX)

S5 Table List of the primers used for the real-time PCR analysis of the carotenoid pathway genes. (XLSX)

S6 Table Carotenoid composition of different potato genotypes over two different growth seasons, determined by LC-PDA-HRMS. Data are the average ± stdev of 3 biological replicates. Asterisks indicate significant variation in an ANOVA plus Tukey's t-test (*: p ≤ 0.05;**: p ≤ 0.01). nd: not detectable.
(XLSX)

S7 Table Identification of xanthophyll mono- and di-esters and their levels, measured by LC-APCI-HRMS. Esters were identified on the basis of their PDA spectra, accurate mass and chemical formula, calculating xanthophylls mono-/di- esters accurate masses, using a mass error < 2ppm. RT, Retention Time; Abs, absorption at λmax nanometers (nm). Ion intensities are expressed as fold Internal standard (ISTD,α-tocopherol-acetate) and values are the avg ± stdev from 3 Technical replicates. ND: not detectable.
(XLSX)

S8 Table Fold change of genes of the carotenoid pathway in the potato core collection, measured by qPCR. Data are expressed as fold change using cv. Daifla as calibrator (FC = 1.000). Data are the average of at least two replications.
(XLSX)

S9 Table Identification and levels of non-polar metabolites in potato genotypes, measured by LC-APCI-HRMS. Non polar metabolites were identified on the basis of their accurate mass (Detected mass, m/z). Ion intensities are expressed as fold Internal standard. Data are the avg ± stdev from 3 biological replications. RT: Retention Time (min); nd: not detectable. Asterisks indicate significant variation in an ANOVA plus Tukey's t-test (*: p ≤ 0.05;**: p ≤ 0.01). nd: not detectable.
(XLSX)

S10 Table Identification and levels of semi-polar metabolites in potato genotypes, measured by LC-ESI-HRMS. Semi-polar metabolites were identified on the basis of their accurate mass (Detected mass, m/z). Ion intensities are expressed as fold Internal standard. Data are the avg ± stdev from 3 biological replications. RT: Retention Time (min); nd: not detectable. Asterisks indicate significant variation in an ANOVA plus Tukey's t-test (*: p ≤ 0.05;**: p ≤ 0.01). nd: not detectable.
(XLSX)

S11 Table Tuber dormancy assessment on potato genotypes. Values were measured on 150 tubers per genotype (3 reps with 50 tubers each), stored in the dark at room temperature: evaluation at sight (100 DAH). Scale: 1–2.9 = very short to short dormancy; 3–5.9 = short to medium-long dormancy; 6–7,9 = medium-long to long dormancy; 6–7,9 = medium-long to long dormancy (National Institute of Agricultural Botany, Cambridge, UK; Netherlands Potato Consultative Foundation, Den Haag, NL).
(XLSX)

S12 Table Potato tuber weight loss. Tubers were weighted twice: at the beginning of storage and after 100 days when dormancy was visually assessed. Weight loss during postharvest storage was expressed as a percentage of initial weight according to the following formula: % of weight loss: (W0 –W100)/W0 x 100 where W0 = initial weight and W100 = weight after 100 days of storage.
(XLSX)

S13 Table Matrix correlation analysis of post-harvest traits, metabolic and gene expression levels in tubers. (XLSX)
